# Assessment of Three-Dimensional Reconstruction in Percutaneous Nephrolithotomy for Complex Renal Calculi Treatment

**DOI:** 10.3389/fsurg.2021.701207

**Published:** 2021-10-20

**Authors:** Haotian Tan, Yaqi Xie, Xuebao Zhang, Wenting Wang, Hejia Yuan, Chunhua Lin

**Affiliations:** ^1^Department of Urology, The Affiliated Yantai Yuhuangding Hospital of Qingdao University, Yantai, China; ^2^Department of Urology, The Affiliated Yantai Yuhuangding Hospital of Binzhou Medical University, Yantai, China; ^3^Department of Reproductive Medicine, The Affiliated Yantai Yuhuangding Hospital of Qingdao University, Yantai, China; ^4^Department of Central Laboratory, Affiliated Yantai Yuhuangding Hospital of Qingdao University, Yantai, China

**Keywords:** complex kidney calculi, efficacy, percutaneous nephrolithotomy, three-dimensional reconstruction, computed tomography

## Abstract

**Introduction:** Three-dimensional (3D) reconstruction is a novel imaging technique widely used to improve surgical operations. Some studies have identified its role in Urology for percutaneous nephrolithotomy (PCNL).

**Objective:** To explore the potential benefits of 3D reconstruction technology in PCNL for complex renal calculi treatment.

**Methods:** A retrospective study involving 139 patients with complex kidney stones who underwent PCNL was conducted between September 30, 2018, to September 30, 2019. Group A patients (72) underwent the 3D reconstruction technique before PCNL, while group B (67) did not. The operation time, the duration of the hospital stay, the puncture accuracy, the decrease in hemoglobin concentration, the stone clearance rate, and the postoperative complications were noted and compared between the two groups.

**Results:** The initial stone clearance rates 2 weeks after PCNL were 81.9 and 64.2% in groups A and B, respectively (*P* < 0.05). The first-time puncture success rates were 87.5 and 47.8 % in groups A and B, respectively (*P* < 0.05). Group A had a shorter operation time than group B (62 vs. 79 min, *P* < 0.05). Besides, the 3D reconstructive technique-assisted patients (91.7%) had no or mild complications, compared with (74.6%) group B patients. There was no significant difference in hemoglobin decline and hospital stay between the two groups.

**Conclusions:** The 3D reconstruction technology is an effective adjunct to PCNL in the complex renal calculi treatment.

## Introduction

Renal stone disease, such as nephrolithiasis or urolithiasis, is a common urinary disease. It has a prevalence rate of 1–5% in Asia (1), and the rates tend to increase. Currently, about 5.8% of adults have renal calculi in China (2). Metabolic bone disease (MBD), chronic kidney disease (CKD), and end-stage renal disease (ESRD) are associated with renal stone disease (3).

Percutaneous nephrolithotomy (PCNL) has been the standard treatment for complex renal stones since 1955 (4, 5). PCNL improves the stone-free rate (6–8). Localization and accurate puncture are essential to PCNL and directly impact the success of the surgery (9, 10). Real-time ultrasound localization is commonly used in clinical practice. However, it is difficult to accurately select the puncture path using the two-dimensional intraoperative ultrasound or X-ray images to treat complex renal calculi, such as calculus in kidney with congenital anomaly and staghorn stones. The shape and the actual calculi position are indistinct, increasing the risk of extra injuries during the operation. Therefore, such operations have higher complication risks (11). In recent years, three-dimensional (3D) CT reconstruction has been used to reduce the rate of surgical concomitant injury and maximize operation accuracy. Moreover, this technique is common in liver operations (12) and is rarely reported in kidney operations. Enhanced CT was often used for 3D reconstruction before PCNL in earlier years. Previous studies indicated that preoperative planning of complex stone situations with 3D-CT had a significant impact on operative procedure, resulting in a low number of access punctures (13, 14). Our research aims to use the non-contrast CT which reduces the impact of the contrast agent on the patient's body and reduces the economic pressure of the patient to perform 3D reconstruction.

In this study, the auxiliary effects of the 3D reconstructive technique on the PCNL were retrospectively investigated. This study can help assess the feasibility and applicable value of the 3D reconstruction technique to treat complex renal stones.

## Materials and Methods

### Patients

The Ethics Committee of the Affiliated Yantai Yuhuangding Hospital of Qingdao University (Yantai, Shandong) approved this study (Approval NO. [2018]71). Each patient who underwent the 3D reconstruction signed the informed consent form. Our study is a retrospective study and it was reported according to STROBE Statement. Patients with solitary kidney and a history of open-stone surgery were excluded. A total of 139 patients with complex renal stones were included in this study. The patients were treated *via* the PCNL between September 30, 2018, to September 30, 2019, and we have de-identified all patient details. Partial or complete staghorn calculi, renal calculi with renal calyx neck stenosis or dilation, and renal calculi with abnormal kidney anatomy defined the complex renal calculi. The financial situation, personal wishes, and other factors determined if 3D reconstruction technique was used in patients or not. Therefore, the participants were divided into two groups (A and B) based on whether or not 3D reconstruction was used.

### Approach

A senior surgeon who accomplished the PCNL over 200 cases conducted all operations. Before the surgical operation, several tests were conducted on the 139 participants, including blood analysis, urine culture, urine analysis, kidney function, and coagulation profile. Imaging examination involving ultrasonography and CT scan was used to determine the stone characteristics. Patients in both groups were received non-contrast CT scan before operation, the radiation dose required to perform 3D reconstruction is ~10 mSv, which is consistent with the dose required for standard preoperative evaluation. Ultrasound-guidance were used intraoperatively to located renal stones. The demographic data of each patient, including age, sex, and body mass index (BMI) were recorded. Clinical indices, such as stone characteristics (stone size, stone type, and stone side), were collected. Besides, the preoperative hydronephrosis perioperative indices, including the operation time, first-time success rate, time to successful puncture, and the change in hemoglobin concentration, were recorded. Postoperative indices recorded, included the hospital stay duration, initial stone clearance rate, final stone clearance rate, and classification of complications were noted.

Stone size was measured at maximal diameter on non-contrast computed tomography scan of the abdomen to reduce measurement error. Each stone was measured three times by three different radiologists and the mean value was taken. The initial and final stone-free rates, were evaluated at 2 and 12 weeks post-operatively *via* the CT scan. Stone-free status was defined as no residual stones in the kidneys or residual fragments sized <3 mm. The modified Clavien grading system was used to evaluate the complication.

### PCNL Preparation

**Figure 1:**
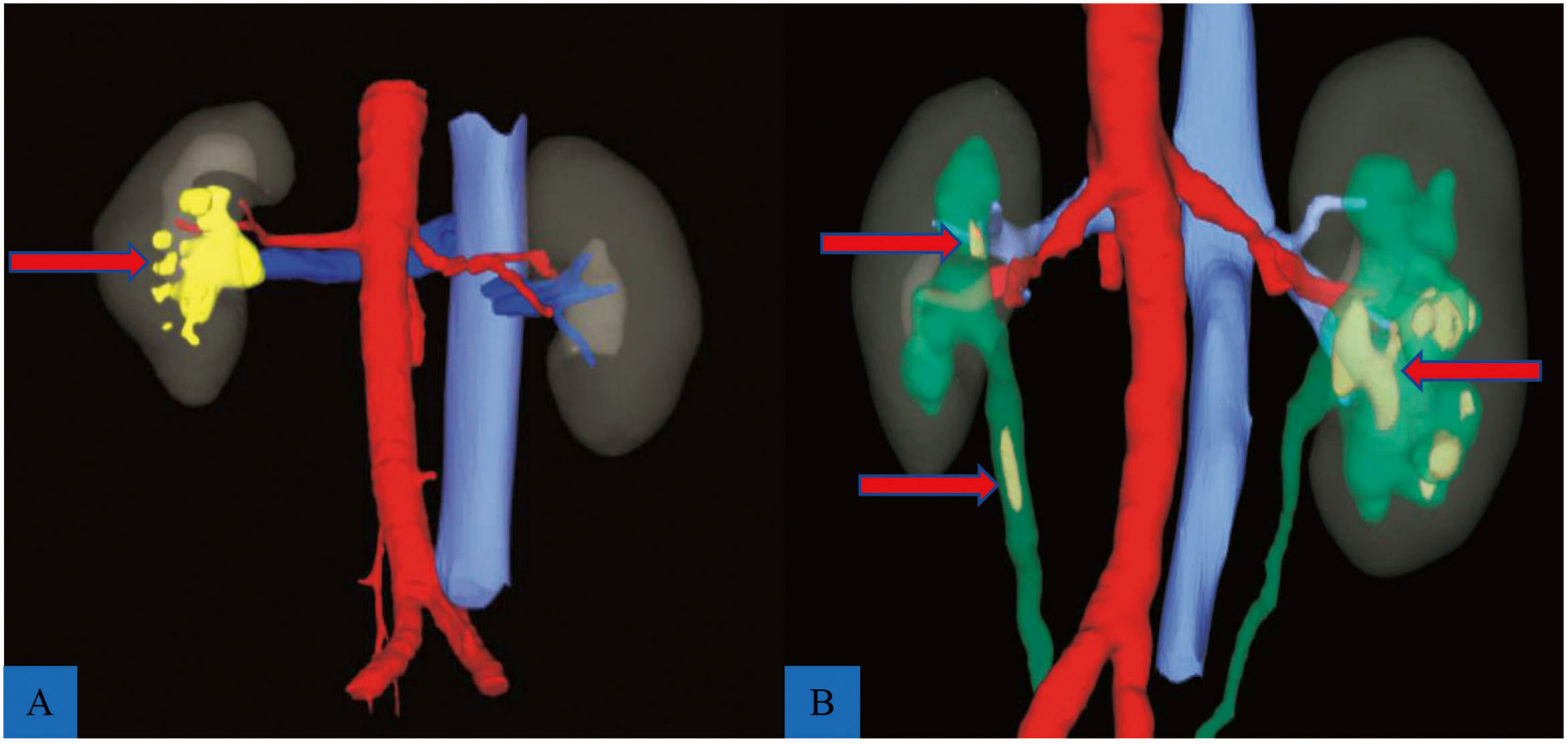
Reconstructed models of two complex renal calculi cases. **(A)** A staghorn stone (red arrow) located in the right kidney; red indicates artery, blue shows vein, and yellow indicates stone. **(B)** Multiple renal calculi (red arrow) complicated with ureteral calculi (red arrow); green shows renal pelvis and ureter.

**Figure 2:**
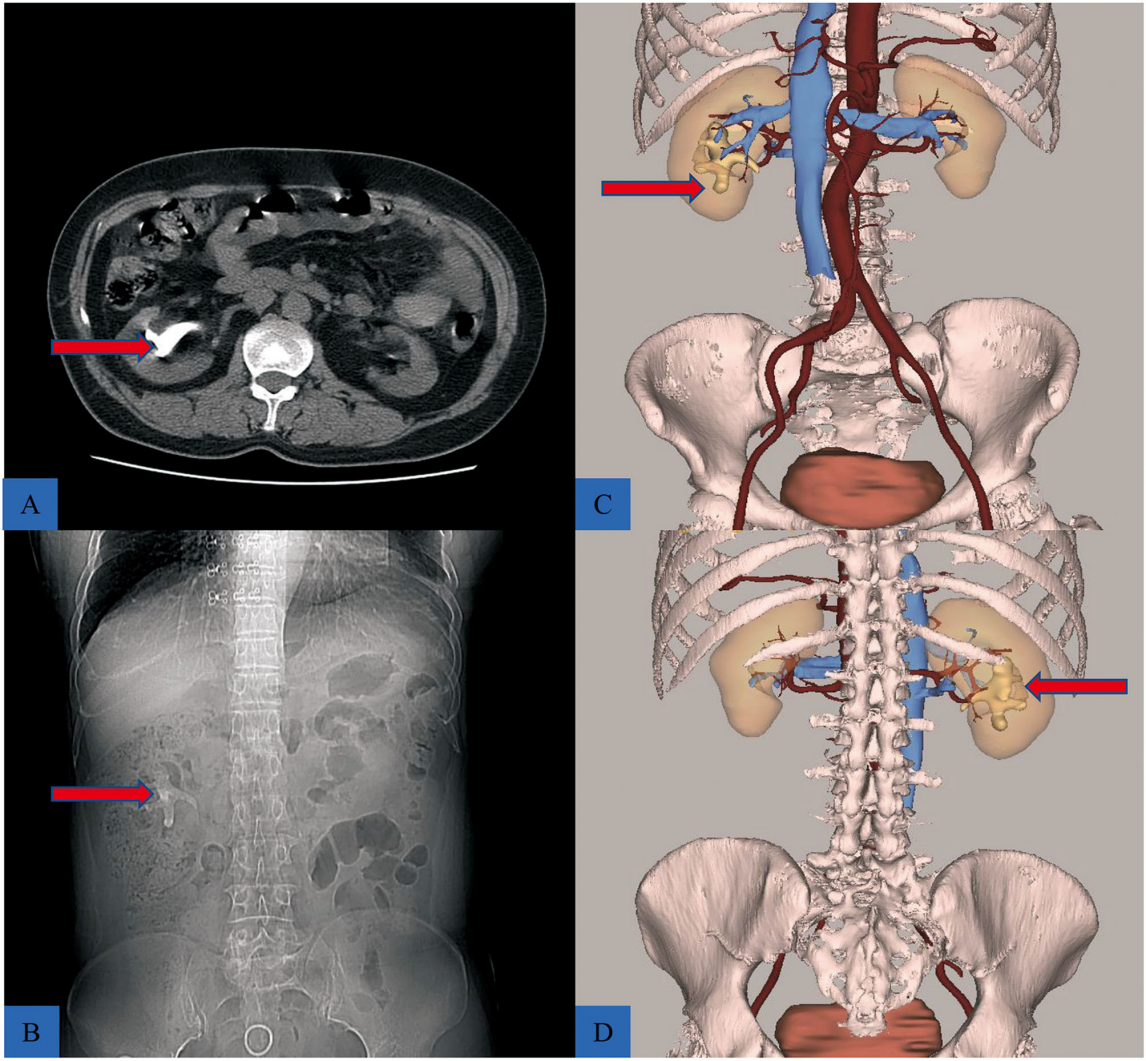
The difference between traditional CT scans, and 3D reconstructive CT scans. **(A,B)** The CT scan showing a staghorn stone (red arrow). **(C)** The front view of reconstructive urinary system including calculi (red arrow), kidney, collective system, and blood vessels, hiding the ureter to better reveal the location of the stone. **(D)** Posterior view of the puncture location, during PCNL.

The 3D reconstructive technique (CAS; Hisense Technology, Qingtao, CN) was used to thoroughly depict the stones in the coronal, vertical, and transverse sections of patients who received 3D reconstruction. The 3D reconstructive CT scans clearly showed the stone's contour indicating the precise calculi location compared to the traditional CT scans (Figure 1). Furthermore, the 3D reconstructive technique showed the relationship between stone, kidney, and collection system (Figure 2), enabling surgeons to evaluate kidney stones from various angles and providing more information on anatomical structures.

### Statistical Analysis

SPSS 25 software was used for all statistical analysis. All continuous variables were represented as the median and interquartile range (IQR) and categorical variables as the number of cases (no) and percentage (%). The Pearson χ2 correlation was used to analyze the significance between categorical variables. Besides, the Mann-Whitney U test was used to compare the significance of continuous data. Statistical significance was set at *P* < 0.05.

## Results

### Patient Characteristics

A total of 139 patients successfully underwent PCNL. Group A patients (72) received the 3D reconstruction technique, while group B (67) did not. The median ages were 57 and 59 years in groups A and B, respectively (*P* = 0.16). Males were 70.8 and 61.2% in group A and B, respectively (*P* = 0.89). The median stone size was not significantly different between the two groups [3.3 cm (IQR 2.6–5.2 cm) and 2.9 cm (IQR 2.5–3.7 cm) in groups A and B, respectively] (*P* = 0.43). Furthermore, eight patients had partial staghorn calculi, and 17 had complete staghorn calculi in group A. Nine and 14 patients, respectively had partial and complete staghorn in group B. There was no significant difference in stone location (*P* = 0.24) and the preoperative hydronephrosis between the two groups (*P* = 0.91). The detailed patient characteristics are shown in Table 1.

**Table 1:** Demographic information of the patients.

**Variable**	**Group A**	**Group B**	***p*** **-value**
No. of patients	72	67	
Age (years), median (IQR)	57 (31–68)	59 (44–70)	0.16
Male, *n* (%)	51 (70.8%)	41 (61.2%)	0.89
BMI (kg/m^2^), median (IQR)	23.38 (17–33)	24.16 (20–32)	0.11
Stone status			
Stone size (cm), median (IQR)	3.29 (2.6–5.2)	2.97 (2.5–3.7)	0.43
Partial staghorn stone, *n* (%)	19 (26.4%)	24 (35.8%)	0.76
Complete staghorn stone, *n* (%)	27 (37.5%)	29 (43.3%)	
Other type of complex renal calculi, *n* (%)	26 (36.1%)	14 (20.9%)	
Location of stones			0.24
Left side, *n* (%)	31 (43.1%)	35 (52.2%)	
Right side, *n* (%)	41 (56.9%)	32 (47.8%)	
Preoperative hydronephrosis			0.91
No or mild level, *n* (%)	43 (59.7%)	51 (76.1%)	
Middle or severe level, *n* (%)	29 (40.3%)	16 (23.9%)	

*BMI, body mass index*.

### Perioperative Data

No intraoperative complications occurred in all the patients, and none required blood transfusion. The 3D reconstructive technique-assisted comprehensive PCNL planning was conducted. The puncture point and direction were planned before PCNL. The median puncture time in group A was significantly shorter than in group B (7.7 vs. 13.3 min, *P* = 0.02). Additionally, most group A patients (87.5%, 63/72) successfully penetrated the required calyx in a single attempt, significantly higher than in group B (47.8%, 32/67), the differences were statistically significant (*P* = 0.03). The operation time was shorter in group A than group B [62 min (IQR 39–131 min) vs. 79 min (IQR 42–s 146 min)] (*P* = 0.01). The initial stone clearance rates in group A were 81.9% (59/72) and 64.2% (43/67) in group B (*P* = 0.01). The final stone clearance rates in group A were 90.3% (65/72) and 83.6% (56/67) in group B (*P* = 0.01). Five patients in group A underwent the second PCNL using the same puncture channel, and two went through shock wave lithotripsy, eight patients in group B underwent the second PCNL and three through shock wave lithotripsy (Table 2).

**Table 2:** The intra- and postoperative clinical outcome.

	**Group A**	**Group B**	***p*** **-value**
Accuracy of puncture			
Time to successful puncture (min), median (IQR)	7.7(3–14)	13.3 (5–28)	0.02
First-time success rate	87.5% (63/72)	47.8% (32/67)	0.03
Operative outcome			
Operating time (min), median (IQR)	62 (39–131)	79 (42–146)	0.01
Decrease in hemoglobin (g/L), median (IQR)	12.5 (7–32)	14.7 (8–34)	0.09
Initial stone clearance rate	81.9% (59/72)	64.2% (43/67)	0.01
Final stone clearance rate	90.3% (65/72)	83.6% (56/67)	0.02
Length of hospital stays (day), median (IQR)	6 (3–13)	7 (3–14)	0.14
Classification of complications			0.03
Grade 0, *n* (%)	55 (76.4%)	34 (50.7%)	
Grade I, *n* (%)	11 (15.3%)	16 (23.9%)	
Grade II, *n* (%)	4 (5.6%)	13 (19.4%)	
More than grade II, *n* (%)	2 (2.7%)	4 (6.0%)	

The 3D reconstruction technique reduced the incidence of postoperative complications. Most group A patients (91.7%, 66/72) had no (grade 0) or mild complications (grade I), significantly higher than in group B (74.6%, 50/67). Grade II complications occurred in both group A (*n* = 4) and group B (*n* = 13). Four group B patients underwent endoscopic invention for ureteric obstruction treatment (class III complication). The decrease in hemoglobin concentration and hospital stay duration was not significantly different between the two groups (less in group A). Table 2 shows the detailed perioperative data.

## Discussion

PCNL is the gold standard and the best surgical approach for complex renal calculi treatment. Several multi-center retrospective studies have shown that PCNL has a higher stone clearance rate and less trauma than traditional surgery, PCNL is recommended in the EUA guidelines as the treatment of staghorn calculi, and in addition, PCNL is also the preferred option for treating the larger stones in the lower pole (6, 15–17). However, PCNL is technically challenging and prone to perioperative complications, such as bleeding and damage to adjacent anatomy, if an unsuccessful puncture or improper dilation occurs (6, 18). Therefore, it is necessary to establish an appropriate working route for PCNL to easily reach the target renal calyx without damaging important blood vessels or adjacent tissues. Puncture accuracy and stability, which is the most critical stage for the operation, determine the appropriate route (19, 20). Therefore, advanced imaging techniques are necessary for preoperative preparation. PCNL has been conducted *via* IVU, renal tract ultrasonography. Besides, El-Wahab et al. recently reported that preoperative multislice CT is a safer, more accurate, and non-invasive imaging technique for mapping the pelvicalyceal system than IVU (21). Nevertheless, 2D CT scans, ultrasound, and X-ray can only provide two-dimensional, lower-resolution, and incomplete critical anatomical structure images. These 2D images result in a long time for preoperative preparation, and increases the risk of operation and anesthesia. Furthermore, some stones could be adjacent to tiny secluded intrarenal blood vessels easily ignored and injured during surgery, causing hemorrhagic shock after surgery. Therefore, a detailed preoperative imaging examination is required to help surgeons choose the ideal puncture scheme.

In recent years, the 3D reconstruction technique has attracted a lot of attention. He et al. indicated that 3D reconstruction technology is effective in preoperative assessment and operation planning of hepatic alveolar echinococcosis (Hae) (22). Türk et al., *via* the international guidelines, reported that contrast-enhancement studies, especially Computed Tomography, are recommended before stone management since it enables the 3D reconstruction of the collected system (17). Thiruchelvam et al. showed that the modified multidetector computed tomographic urography (CTU) technique could be used for patients with complex pelvicalyceal anatomy but at increased radiation exposure cost (23). Furthermore, the CTU is time-consuming, expensive, not universally available, and is a radiological technique with high radiation dose. Previous reports have shown that 3D CT scan reconstruction is noninvasive, cost-effective with high-quality 3D images of renal calculi (24–26). Therefore, 3D reconstruction can significantly enhance PCNL.

In this study, group A patients (72) received the 3D reconstructive technique. The operation time and the incidence of postoperative complications were significantly reduced in group A, indicating that 3D reconstruction may provide more effective technical assistance in complex renal calculi treatment requiring high precision puncture. The 3D reconstructive renal stone images aided comprehensive PCNL planning. Therefore, the renal calyx puncture was accurate, with the initial and final stone clearance rates in groups A are higher than those of group B. Additionally, the 3D reconstructive technique improved the puncture accuracy as estimated *via* two markers (time to successful puncture and the first-time success rate). The first-time success rates were significantly different between the two groups. Moreover, the 3D reconstructive technique aids the surgeon to accurately select the target calyceal before surgery and formulate an exact puncture angle and needle depth, reducing the time required for a puncture (Table 2).

Group A patients had advanced 3D reconstruction images before the operation, while those in group B had CT KUB. Tailly et al. reported that advanced techniques such as 3D reconstruction technology provide no added value compared to simple manual measurements of stone burden with respect to stone-free status. Nevertheless, Tailly et al. indicated that 3D reformatting of CT images could provide a more accurate stone burden measure than one or two-dimensional measures and valuable information for PCNL planning (27), consistent with the study results.

A decrease in hemoglobin after PCNL is common. Recently, several clinical studies have shown that the occurrence of blood transfusions caused by PCNL varies widely, from 1 to 5% (28–30). Previous studies showed that 1–12% require a blood transfusion after PCNL (31, 32). Several recent studies have demonstrated that the blood transfusion rate is <2.5% (33), and only 3% of patients experience severe bleeding with symptoms, such as hypotension, shock, or renal insufficiency (34, 35). In this study, while the length of hospital stays and the hemoglobin decrease was less in group A than in group B, the difference was not statistically significant. Therefore, it is necessary to use a large study to verify if the differences between the groups are substantial. The reduced operation time caused by the experience and specialization of the surgical team also affects the incidence of complications.

The 3D reconstructive CT scans of renal stones provide more information on the overall operation planning, thus improving the success rate and safety of PCNL. This study demonstrates the effectiveness of using 3D reconstruction techniques in patients with kidney stones with renal anatomical structure variation and all complex kidney stones, to choose the optimal percutaneous approach to reduce the operation time and improve safety. Although this technique does not amend the standard patient position, it is cost-effective and can significantly improve preoperative understanding of stone location, size, shape, and orientation *via* the 3D models compared to CT alone. Notably, the 3D viewing of the anatomy significantly improved preoperative understanding of the optimal calyx of entry. Furthermore, the 3D reconstruction technique improved patients' knowledge of stone disease and reduced their preoperative anxiety. Importantly, a 3D renal stone database can be developed to train urologist residents in PCNL due to the increased 3D images of complicated renal calculi reconstructed using this new method.

However, this study has some limitations. The nature of retrospective design and analysis was the major limitation. This research was non-randomized. The study also used limited cases from a single medical center, thus the statistical analysis of data could be biased. Background differences cannot be fully controlled and can affect results.

Further larger and longer-term studies are required to investigate surgical outcomes. Nevertheless, 3D reconstruction can be novel and effective in managing complex renal stones. In this experiment, 3D reconstruction has been shown to shorten the operation time, improve the stone-free rate, improve the success rate of puncture, and the first puncture and reduce the puncture time.

## Conclusion

The 3D reconstructive technique can help obtain comprehensive information before PCNL. Besides, it provides technical feasibility for the comprehensive planning of patients with complex kidney stones. It can minimize the risks associated with the operation and improve the stone clearance rate. Therefore, 3D reconstruction technology can be used before PCNL to obtain easy, direct, and safe access, improve stone clearance, and reduce complication rates. However, large, well-conducted studies are needed to verify these results.

## Data Availability Statement

The data that support the findings of this study are available on request from the corresponding author. The data are not publicly available owing to privacy or ethical restrictions.

## Ethics Statement

The studies involving human participants were reviewed and approved by the Ethics Committee of the Affiliated Yantai Yuhuangding Hospital of Qingdao University (Yantai, Shandong). The patients/participants provided their written informed consent to participate in this study.

## Author Contributions

HT and YX analyzed the data and wrote this manuscript. XZ collected and assembled the clinic data. CL and HY designed the study project. WW analyzed and interpreted the data. All authors read and approved the final manuscript.

## Funding

This research was funded by Natural Science Foundation of Shandong Province, Grant/Award Number: ZR2019MH132; Yantai Key Research and Development Project, Grant/Award Numbers: 2018SFGY115 and 2019MSGY135.

## Conflict of Interest

The authors declare that the research was conducted in the absence of any commercial or financial relationships that could be construed as a potential conflict of interest.

## Publisher's Note

All claims expressed in this article are solely those of the authors and do not necessarily represent those of their affiliated organizations, or those of the publisher, the editors and the reviewers. Any product that may be evaluated in this article, or claim that may be made by its manufacturer, is not guaranteed or endorsed by the publisher.

## References

[1] Sorokin I Mamoulakis C Miyazawa K Rodgers A Talati J Lotan Y. Epidemiology of stone disease across the world. World J Urol. (2017) 35:1301–20. 10.1007/s00345-017-2008-6 28213860

[2] Zeng G Mai Z Xia S Wang Z Zhang K Wang L. Prevalence of kidney stones in China: an ultrasonography based cross-sectional study. BJU Int. (2017) 120:109–16. 10.1111/bju.13828 28236332

[3] Parmar MS. Kidney stones. BMJ. (2004) 328:1420–4. 10.1136/bmj.328.7453.1420 15191979 PMC421787

[4] Patel SR Nakada SY. The modern history and evolution of percutaneous nephrolithotomy. J Endourol. (2015) 29:153–7. 10.1089/end.2014.0287 25093997

[5] Al-Kohlany KM Shokeir AA Mosbah A Mohsen T Shoma AM Eraky I. Treatment of complete staghorn stones: a prospective randomized comparison of open surgery versus percutaneous nephrolithotomy. J Urol. (2005) 173:469–73. 10.1097/01.ju.0000150519.49495.88 15643212

[6] Zeng G Zhao Z Wan S Mai Z Wu W Zhong W. Minimally invasive percutaneous nephrolithotomy for simple and complex renal caliceal stones: a comparative analysis of more than 10,000 cases. J Endourol. (2013) 27:1203–8. 10.1089/end.2013.0061 23924320 PMC3787398

[7] Akman T Sari E Binbay M Yuruk E Tepeler A Kaba M. Comparison of outcomes after percutaneous nephrolithotomy of staghorn calculi in those with single and multiple accesses. J Endourol. (2010) 24:955–60. 10.1089/end.2009.0456 20443700

[8] Ozturk U Sener NC Goktug HG Nalbant I Gucuk A Imamoglu MA. Comparison of percutaneous nephrolithotomy, shock wave lithotripsy, and retrograde intrarenal surgery for lower pole renal calculi 10-20 mm. Urol Int. (2013) 91:345–9. 10.1159/000351136 23816573

[9] Watterson JD Soon S Jana K. Access related complications during percutaneous nephrolithotomy: urology versus radiology at a single academic institution. J Urol. (2006) 176:142–5. 10.1016/S0022-5347(06)00489-7 16753389

[10] Mandal S Goel A Goyal NK. Re: staghorn morphometry: a new tool for clinical classification and prediction model for percutaneous nephrolithotomy monotherapy: (From: Mishra S, Sabnis RB, Desai M. J Endourol 2012; 26: 6–14). J Endourol. (2012) 26:1099. 10.1089/end.2012.0088 22050495

[11] Xu Y Wu Z Yu J Wang S Li F Chen J. Doppler ultrasound-guided percutaneous nephrolithotomy with two-step tract dilation for management of complex renal stones. Urology. (2012) 79:1247–51. 10.1016/j.urology.2011.12.027 22365450

[12] Radtke A Nadalin S Sotiropoulos G Molmenti E Schroeder T Valentin-Gamazo C. Computer-assisted operative planning in adult living donor liver transplantation: a new way to resolve the dilemma of the middle hepatic vein. World J Surg. (2007) 31:175. 10.1007/s00268-005-0718-1 17180479

[13] Park S Pearle MSJUC. Imaging for percutaneous renal access and management of renal calculi. Urol Clin North Am. (2006) 33:353–64. 10.1016/j.ucl.2006.03.003 16829270

[14] Thiruchelvam N Mostafid H Ubhayakar G. Planning percutaneous nephrolithotomy using multidetector computed tomography urography, multiplanar reconstruction and three-dimensional reformatting. BJU Int. (2005) 95:1280–4. 10.1111/j.1464-410X.2005.05519.x 15892817

[15] Abdelhafez MF Bedke J Amend B ElGanainy E Aboulella H Elakkad M. Minimally invasive percutaneous nephrolitholapaxy (PCNL) as an effective and safe procedure for large renal stones. BJU int. (2012) 110:E1022–6. 10.1111/j.1464-410X.2012.11191.x 22540846

[16] Knoll T Daels F Desai J Hoznek A Knudsen B Montanari E. Percutaneous nephrolithotomy: technique. World J Urol. (2017) 35:1361–8. 10.1007/s00345-017-2001-0 28124111

[17] Türk C Skolarikos A Neisius A Petřík A Seitz C Knoll T. EAU Guidelines on Urolithiasis. Available online at: http://uroweb.org/guideline/urolithiasis (accessed May 6, 2018).

[18] Rosette JDL Assimos D Desai M Gutierrez J Lingeman J Scarpa R. The clinical research office of the endourological society percutaneous nephrolithotomy global study: indications, complications, and outcomes in 5803 patients. J Endourol. (2011) 25:11–7. 10.1089/end.2010.0424 21247286

[19] Yan S Xiang F Yongsheng S. Percutaneous nephrolithotomy guided solely by ultrasonography: a 5-year study of >700 cases. BJU int. (2013) 112:965–71. 10.1111/bju.12248 23889729

[20] Lu MH Pu XY Gao X Zhou XF Qiu JG Jie ST. A comparative study of clinical value of single B-Mode ultrasound guidance and B-Mode combined with color Doppler ultrasound guidance in mini-invasive percutaneous nephrolithotomy to decrease hemorrhagic complications. Urology. (2010) 76:815–20. 10.1016/j.urology.2009.08.091 20579695

[21] El-Wahab OA El-Tabey MA El-Barky E El-Baky SA El-Falah A Refaat M. Multislice computed tomography vs. intravenous urography for planning supine percutaneous nephrolithotomy: a randomised clinical trial. Arab J Urol. (2014) 12:162–7. 10.1016/j.aju.2013.11.005 26019942 PMC4434608

[22] He Y-B Bai L Aji T Jiang Y Zhao J-M Zhang J-H. Application of 3D reconstruction for surgical treatment of hepatic alveolar echinococcosis. World J Gastroenterol. (2015) 21:10200. 10.3748/wjg.v21.i35.10200 26401085 PMC4572801

[23] Ghani KR Patel U Anson K. Planning percutaneous nephrolithotomy using multidetector computed tomography urography, multiplanar reconstruction and three-dimensional reformatting. BJU int. (2005) 96:916–7. 10.1111/j.1464-410X.2005.05841_3.x 16153231

[24] Bandi G Meiners RJ Pickhardt PJ Nakada SY. Stone measurement by volumetric three-dimensional computed tomography for predicting the outcome after extracorporeal shock wave lithotripsy. BJU int. (2008) 103:524–8. 10.1111/j.1464-410X.2008.08069.x 19007365

[25] Finch W Johnston R Shaida N Winterbottom A Wiseman O. Measuring stone volume - three-dimensional software reconstruction or an ellipsoid algebra formula? BJU int. (2014) 113:610–4. 10.1111/bju.12456 24053445

[26] Hubert J Blum A Cormier L Claudon M Regent D Mangin P. Three-dimensional CT-scan reconstruction of renal calculi. a new tool for mapping-out staghorn calculi and follow-up of radiolucent stones. Eur Urol. (1997) 31:297–301. 10.1159/000474471 9129919

[27] Tailly T Nadeau BR Violette PD Bao Y Amann J Nott L. Stone burden measurement by 3D reconstruction on noncontrast computed tomography is not a more accurate predictor of stone-free rate after percutaneous nephrolithotomy than 2D stone burden measurements. J Endourol. (2020) 34:550–7. 10.1089/end.2019.0718 32008375

[28] Seitz C Desai M Häcker A Hakenberg OW Liatsikos E Nagele U. Incidence, prevention, and management of complications following percutaneous nephrolitholapaxy. Eur Urol. (2012) 61:146–58. 10.1016/j.eururo.2011.09.016 21978422

[29] Keoghane SR Cetti RJ Rogers AE Walmsley BH. Blood transfusion, embolisation and nephrectomy after percutaneous nephrolithotomy (PCNL). BJU Int. (2013) 111:628–32. 10.1111/j.1464-410X.2012.11394.x 22958458

[30] Senocak C Ozbek R Bozkurt OF Unsal AJU. Predictive factors of bleeding among pediatric patients undergoing percutaneous nephrolithotomy. Urolithiasis. (2018) 46:383–9. 10.1007/s00240-017-1001-2 28702679

[31] Wezel F Mamoulakis C Rioja J Michel MS de la Rosette J Alken P. Two contemporary series of percutaneous tract dilation for percutaneous nephrolithotomy. J Endourol. (2009) 23:1655–61. 10.1089/end.2009.0213 19558265

[32] Agrawal MS Agrawal M Gupta A Bansal S Yadav A Goyal J. A randomized comparison of tubeless and standard percutaneous nephrolithotomy. J Endourol. (2008) 22:439–42. 10.1089/end.2007.0118 18257738

[33] Soucy F Ko R Duvdevani M Nott L Denstedt JD Razvi H. Percutaneous nephrolithotomy for staghorn calculi: a single center's experience over 15 years. J Endourol. (2009) 23:1669–73. 10.1089/end.2009.1534 19715482

[34] Ziaee SA Karami H Aminsharifi A Mehrabi S Zand S Javaherforooshzadeh A. One-stage tract dilation for percutaneous nephrolithotomy: is it justified? J Endourol. (2007) 21:1415–20. 10.1089/end.2006.0454 18186676

[35] El-Assmy AM Shokeir AA Mohsen T El-Tabey N El-Nahas AR Shoma AM. Renal access by urologist or radiologist for percutaneous nephrolithotomy—is it still an issue? J Urol. (2007) 178:916–20. 10.1016/j.juro.2007.05.015 17632136

